# Adverse Events Following Immunization in Brazil: Age of Child and Vaccine-Associated Risk Analysis Using Logistic Regression

**DOI:** 10.3390/ijerph15061149

**Published:** 2018-06-01

**Authors:** Sílvia R.C. Lopes, João L.R. Perin, Taiane S. Prass, Sandra Maria D. Carvalho, Sérgio C. Lessa, José G. Dórea

**Affiliations:** 1 Institute of Mathematics, Federal University of Rio Grande do Sul (UFRGS), Porto Alegre RS 91509-900, Brazil; silvia.lopes@ufrgs.br; 2 Management and Technical Support, StatSoft South America, Porto Alegre RS 90040-190 Brazil; joao_perin32@hotmail.com; 3 Statistics Department, Federal University of Santa Maria (UFSM), Santa Maria RS 97105-900, Brazil; taianeprass@gmail.com; 4 National Immunization Program/SVS/MS, Brasília 70058-900, Brazil; sandra.deotti@saude.gov.br; 5 National Research Council (CNPq), COAGR/CGAPB, Brasília 71605-001, Brazil; sclessa@hotmail.com; 6 Faculty of Health Sciences, University of Brasília, Brasília 70919-970, Brazil

**Keywords:** vaccine, adverse event, immunization, AEFI, passive surveillance

## Abstract

*Objective*: Vaccines are effective in controlling and eradicating infectious diseases. However, adverse events following immunization (AEFI) can occur in susceptible individuals. The objective of this study was to analyze the Brazilian AEFI database and compare eight vaccines in order to profile risks of AEFIs related to the mandated pediatric schedule of immunization, considering the age and sex of the child, type of vaccine, and reported adverse events. *Methods*: We analyzed the Brazilian AEFI database integrating reports between 2005 and 2010 for children less than 10-years old immunized with eight mandated vaccines: diphtheria, pertussis, tetanus, *Haemophilus influenzae* type b (TETRA); diphtheria, tetanus, and pertussis (DTP); Bacillus Calmette–Guerin (BCG); oral poliovirus vaccine (OPV); measles, mumps, and rubella (MMR); oral rotavirus vaccine (ORV); hepatitis B (HB); and yellow fever (YF). We compared the children’s age regarding types of AEFI, evaluated AEFI factors associated with the chance of hospitalization of the child, and estimated the chance of notification of an AEFI as a function of the type of vaccine. In total, 47,105 AEFIs were observed for the mandated vaccines. *Results*: The highest AEFI rate was for the TETRA vaccine and the lowest was for the OPV vaccine, with 60.1 and 2.3 events per 100,000 inoculations, respectively. The TETRA vaccine showed the highest rate of hypotonic hyporesponsive episode, followed by convulsion and fever. The MMR and YF vaccines were associated with generalized rash. BCG was associated with enlarged lymph glands but showed the largest negative (protective) association with hyporesponsive events and seizures. Compared with children aged 5–9-years old, young children (<1 year) showed significantly higher odds of hospitalization. *Conclusions*: The Brazilian AEFI registry is useful to compare the magnitude and certain characteristics of adverse events associated with mandated pediatric vaccines.

## 1. Introduction

Public health policies to control and eradicate infectious diseases depend on the immunization success of specific vaccines. In many parts of the world where medical resources and health infrastructure are lacking, vaccines are a cost-effective public-health intervention [1]. In fact, they are even more cost-effective in monetary terms in rich countries where the expensive treatment of infectious diseases is prevented, freeing up health care resources for purposes other than treating preventable diseases. Furthermore, immunization policies have enabled important achievements in public health, such as the eradication and/or control of yellow fever, smallpox, and poliomyelitis [1].

The protocol to register and/or license vaccines is rigidly controlled in order to achieve and to ensure their safety and effectiveness. Nevertheless, after a vaccine is licensed, susceptible individuals can react to vaccine ingredients (antigens and other constituents in the formulation). Most reactions to vaccines are reported as discomfort, induration at the site of the inoculation, and pain [2]. These occurrences after vaccination are mild and resolve themselves; severe events following vaccination are relatively rare [2]. However, a vaccine surveillance and follow-up system is crucial to monitor issues related to adverse events following immunization (AEFI) [3]. These AEFI systems, as summarized by Zhou et al. [4], are important to “(1) detect new, unusual, or rare vaccine adverse events; (2) monitor increases in known adverse events; (3) determine patient risk factors for particular types of adverse events; (4) identify vaccine lots with increased numbers or types of reported adverse events; and (5) assess the safety of newly licensed vaccines”.

Despite a stringent safety protocol during development, vaccines, like any pharmaceutical product, carry risks. Because of size, clinical trials during vaccine development are not suitable for capturing rare or deferred adverse events/effects [5]. Therefore, essential monitoring of temporal associations between vaccination and the occurrence of serious, mild adverse, or unexpected reactions to vaccination depend on such systems. Infrastructure for reporting AEFIs is in place in many countries, including Brazil [6]. However, these systems and/or appropriate infrastructure are absent in most developing countries, thus leaving the neediest populations without an AEFI vigilance system.

As discussed by Waldman et al. [7], concerns about the safety of vaccines while maintaining high levels of immunization coverage have led countries (with different health care structures) to implement surveillance systems to monitor adverse events postvaccination. These passive surveillance systems are characterized by the capture of spontaneous notification, thus constituting the simplest way of analyzing adverse events.

Because vaccines are administered to large cohorts of healthy children (infants and small children), it is crucial to monitor the burden of AEFIs. Brazil has a successful immunization program, and in 1998, created the systems of vigilance for adverse postvaccination events (SVEAPV) nationwide. In Brazil, AEFIs are collected through the SVEAPV, which can minimize some deficiencies by consistently identifying the type of vaccine, dose, and the subject. We analyzed this database to compare eight vaccines from the mandated pediatric schedule of immunization in order to profile risks of AEFIs related to the age and sex of the child, type of vaccine, and reported adverse events.

## 2. Materials and Methods

This is a descriptive study analyzing the frequency and distribution of the most common AEFI reported in children from 2005 to 2013. The study was approved by the Ethics Committee of the Federal University of Brasilia (UnB CAAE #41787215.5.0000.0030) and consisted of an analysis of the data collected in AEFIs captured by all Brazilian states through the National Immunization Program (PNI). In this study, the data were presented collectively in order to safeguard the integrity and anonymity of all those involved (patients and health care agents). The results were used only for the purpose of the statistical study and not as evidence for or against a specified vaccine. Adverse events here were used to mean “any moderate or severe and/or unexpected adverse sign or symptom occurring after vaccination” [7].

The AEFI database is organized by the Central Office of the PNI after receiving structured information in a standardized form. These data are digitalized and electronically sent to the pharmacovigilance office of the PNI. A detailed account of how the Brazilian AEFI works is provided in Waldman et al. [7] and Monteiro et al. [8].

The database, during the period of 2005–2010, contained the number of AEFIs for children aged less than 10-years old who received any of the eight vaccines available in all public-run (federal, state, or municipal) hospitals or equally public-run health centers throughout the country, free of charge. In parallel, a strong private medical sector provides vaccinations with a cost. Thus, pediatric patients have optional access to private-run hospitals and vaccination clinics which are not bound to report AEFIs.

Most of the recommended vaccines, around 90%, are produced in Brazil [9]. Monteiro et al. [8] have described in detail the passive Brazilian AEFI system. It receives reports prepared by specialized personnel (nurses or physicians) on a regular basis from hospitals, primary health care units, and vaccination clinics throughout the country. The AEFI reports are filled out using a specific form that captures demographic data, dates of vaccination and reported AEFIs, characteristics of the adverse event (type, severity, treatment, and length of hospital stay, whether in- or outpatient), maintenance of the vaccination schedule, as well as the manufacturer of the vaccine and the respective lot number. Monteiro et al. [8] also informed that the completeness of these data ranges from 70.0% to 90.0%, with a trend toward increasing completeness.

Only the events related to a single inoculation were used to summarize the 10 most frequent AEFIs in order to analyze the association between the chosen events and vaccines and to analyze the differences between vaccines regarding the distribution of children’s age. With this approach, we guaranteed that there was no other vaccine causing the adverse reaction under consideration. The total number of adverse events reported after the application of one or more vaccines was used to model the likelihood of hospitalization after the specific AEFI.

In this study, the following vaccines were considered: diphtheria, pertussis, tetanus, meningitis, and other infections caused by *Haemophilus influenzae* type b (TETRA) (three doses); diphtheria, tetanus, and pertussis (DTP) (two doses); Bacillus Calmette–Guerin (BCG) (one dose); oral poliovirus vaccine (OPV) (three doses plus the “booster” shot); measles, mumps, and rubella (MMR) (two doses); oral rotavirus vaccine against diarrhea (ORV) (two doses); hepatitis B (HB) (three doses); and yellow fever (YF) (only one dose every 10 years). Only two of these vaccines (ORV and OPV) are administered orally, while the remaining six vaccines are given through injections. In 2005, the estimated Brazilian population aged less than 10-years old numbered 35,809,635. The vaccine coverage in Brazil ranged from 81% to 85% of children aged 18 months in the very low to medium high socioeconomic classes [10]. Individuals aged 10 or over and those without age recorded were excluded. Because of convenience and availability, only the years from 2005 to 2010 were analyzed.

In the statistical analysis, we considered the following variables (and respective categories) for any child with a reported AEFI: age of child (<1-year old; 1–4-years old; 5–9-years old (reference group); sex (boys and girls as the reference group); regions by state (27); year of report (2005–2010); vaccine type (DTP, BCG, OPV, MMR, ORV, HB, and YF; TETRA is the reference vaccine); AEFI (73 reported types); hospitalization (yes or no used as the reference groups); vaccine dose (first dose, second dose, and third dose; first booster and second booster as the reference groups).

### Statistical Analysis

For the adjustments of the database and all the statistical analyses considered in this work, we used the free software R (see the R-project in https://www.r-project.org). We considered the chi-squared and Kruskal–Wallis hypothesis tests and adjusted residuals together with the Dunn hypothesis test (package *pgirmess*) for multiple comparisons. We also used this software for both the unconditional and correlated logistic regression analyses. The correlated logistic regression, also known as generalized estimating equations (GEE), was run with the *gee* R-package.

The homogeneity chi-squared hypothesis test was used to obtain the statistical significance between eight independent vaccine types related to the 73 reported AEFI types (H^1^__0_: *there was homogeneity among vaccine types as to the AEFI distribution*). The analysis of the adjusted residuals is recommended whenever the null hypothesis H^1^__0_ is rejected. The Kruskal–Wallis hypothesis test was used to decide if there was any difference among the eight independent vaccine types related to the age of any child who suffered any reported AEFI (H^2^__0_: *there was no difference among the ages of children in all eight vaccine types*). When the null Kruskal–Wallis hypothesis test was rejected, we used the Dunn hypothesis test for a multiple comparison analysis. The methodology for all hypothesis tests is found elsewhere [11,12].

To investigate the association between the hospitalization rate and the reported AEFI in a child, we considered the logistic regression analysis: the effect of *p* independent random variables X over a dichotomous dependent random variable Y (Y = 1, meaning that a *hospitalization event occurs* while Y = 0 means that *no hospitalization event occurs*). In the logistic regression analysis, we used both the univariate and multivariate schemes. In the univariate case, we analyzed the odds ratio of the hospitalization groups based on a reference category for each variable, while in the multivariate case, we used the same analysis but now took into account the effects of all other variables.

We also considered both the uncorrelated and correlated data. The analytic approach used for modeling the outcome variables that had dichotomous correlated responses was the generalized estimating equations (GEE). This method took into account the correlated nature of the responses. We refer the reader to Hosmer and Lemeshow [13] and Kleinbaum and Klein [14] for the logistic regression analysis.

## 3. Results

A total of 47,105 AEFIs records captured by the Brazilian National Immunization System were analyzed and recorded according to single or multiple inoculations on a given visit during the studied period of 2005–2010. This total pertained to records of patients that needed medical attention after an AEFI (first or second notification). Overall descriptive results are summarized in Table 1, Table 2 and Table 3 by age and type of vaccine.

Table 1 shows the total number of AEFIs associated with the eight studied vaccines. Additionally, when more than one mandated vaccine was applied on the same visit, there were AEFIs for the specified vaccine. A total of 36,953 adverse cases were associated with 36,742 children. Therefore, 211 adverse events were associated with children who had already experienced AEFIs (second notification) from a previous immunization. For each notification, more than one adverse event could have occurred. Therefore, there was an average of 1.28 AEFIs per child. The ratio of single and combined inoculations (SI/CI in Table 1) shows the proportion of adverse events attributed to a single vaccine as a function of the total AEFI reported for that vaccine.

The frequencies of the 10 most reported AEFIs are presented in Table 2 and Table 3 as injectable and oral vaccines, respectively. Table 2 summarizes AEFIs for injected vaccines by age groups. The 10 most reported events corresponded to 90.24% (27,357) of the total. For all injectable vaccines, the most reported adverse event was hypotonic hyporesponsive episode (HHE), accounting for 33.32% (9115) of all the AEFIs, and it occurred mainly in the inactivated vaccines (TETRA and DTP). The tetravalent vaccine presented the highest number of notifications among the injectable vaccines, with 30,315 events occurring after a single application. For the TETRA vaccine, the age range “less than 1-year old” accounted for 90.57% (24,776) of the 10 most reported events (see Table 2). For the DTP vaccine, the 10 most reported AEFIs accounted for 87.70% (5489) of the total. Among them, “pain, redness, and warmth” (local reaction) accounted for 23.81% (1307) of the events (Table 2). The most frequent DTP-related AEFI usually occurred in age class “1–4-years old”, with 70.41% (3865) of the events. The AEFIs associated with HB vaccine corresponded to 79.98% (747) of the total number of events reported, and “hot spot abscess” was the most reported adverse event, with 28.32% (219). The HB-related AEFIs occurred mostly in children younger than 1-year old, with 96.25% (719) of all notifications (Table 2).

Among the live attenuated vaccines (BCG, MMR, YF), AEFIs after BCG corresponded to 92% (3222) of the total. The most commonly reported AEFI was “nonsuppurative lymphadenopathy”, with 24.46% (788) of the events. The most frequent BCG-related AEFI occurred mostly in children younger than 1-year old, comprising 72.16% (2846) of the notifications. The BCG vaccine was responsible for 3501 adverse events after a single application, with 97.4% of all reactions possibly associated with it (Table 1). AEFIs occurring with the MMR vaccine corresponded to 86.7% (1114) of the total reported events. The most reported adverse event for this vaccine was “generalized rash”, with 41.47% (462) of the events. The MMR-AEFI occurred mostly in children in the group “1–4 years-old”, with 84.56% (942) of the notifications. For the application of the YF vaccine alone, the 10 most frequent events corresponded to 81.83% (725) of all notifications. According to Table 2, the most prevalent was “generalized rash”, with 24.41% (177) of the adverse events. Most YF-related AEFIs occurred in children younger than 1 year of age, corresponding to 70.34 % (510) of the total notifications.

The AEFIs related to the oral vaccines (OPV and ORV) are shown in Table 3. Overall, the oral vaccines showed the lowest rate of AEFIs, that is, 43.2% and 21.6%, respectively (Table 1).

However, for the ORV vaccine alone, the event with the highest number of notifications was “other serious or unusual events”, with 48.68% (332) of the total, followed by “intussusception”, with 25.95% (177) of total notifications (Table 3). The AEFIs occurred mostly in children younger than 1 year of age, corresponding to 98.53% (672) of the total events (Table 3).

**Figure 1:**
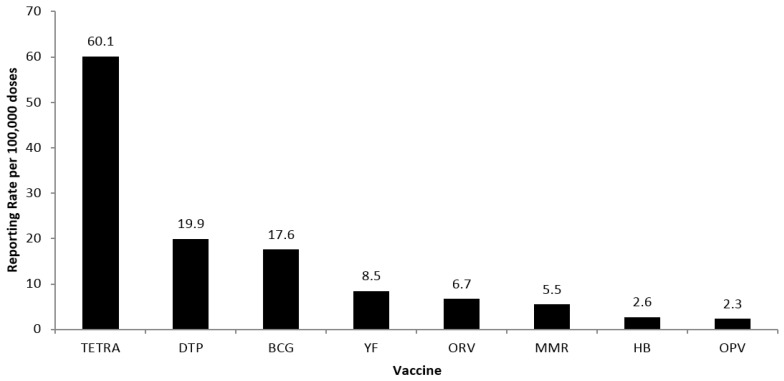
Estimated rate of AEFI per 100,000 doses in children younger than 10-years old, between 2005 and 2010 considering single and combined inoculations (TETRA: diphtheria, pertussis, tetanus, Haemophilus influenzae type b; DTP: diphtheria, tetanus, pertussis; BCG: Bacillus Calmette–Guerin; MMR: measles, mumps, rubella; HB: hepatitis B; YF: yellow fever).

Overall, most of the reported AEFIs (75%) occurred in children less than 1 year of age (35,393), followed by children in the age group between 1 and 4 years of age (9555). Older children in the age group of 5–9 years of age showed the lowest number of cases (2157). However, the median age for AEFI varied according to vaccines, reflecting the recommended immunization calendar as follows: DTP (19 months), OPV (16 months), MMR (13 months), YF (10 months), TETRA (5 months), ORV (4 months), BCG (3 months), and HB (2 months). As a function of the total number of doses of vaccine, the rate of AEFIs varied from 60.1 (TETRA) to 2.3 (OPV) per 100,000 doses, as illustrated in Figure 1.

Based on the adjusted residuals, it is possible to note the most significant associations. After a homogeneity chi-squared hypothesis test, Appendix A Table A1 shows the adjusted residuals for the estimates of the association between vaccines and AEFIs. Table A1 shows that among the 10 most reported adverse events, the most significant positive association occurred between the TETRA vaccine and HHE, with fever without convulsions and afebrile and febrile convulsion events. Regarding the other featured events, the vaccines MMR and YF were significantly associated with a generalized rash event. The BCG vaccine showed the largest negative association (protective) with HHE event, with an adjusted residual of −37.6, while the BCG vaccine’s largest positive association occurred with increased lymph nodes, with an adjusted residual of 80.2.

The Kruskal–Wallis hypothesis test showed that there is statistically significant evidence that the median age of affected children is different for at least one vaccine. The results of a multiple comparison Dunn hypothesis test are shown in Appendix A Table A2. At a 5% significance level, the DTP vaccine had a significantly higher median age than all the other vaccines when considering affected children with some kind of AEFI after a single inoculation. The HB vaccine showed the highest association, with AEFI in children with the lowest median age, that is, two months. The OPV, MMR, and YF vaccines showed no significant differences. Likewise, the test showed no significant differences between ORV and BCG vaccines where the median ages were, respectively, four and three months. AEFI events after the TETRA vaccine inoculation occurred in children with a median age of five months old, which is significantly higher than the median age for ORV, BCG, and HB vaccines.

When considering both the univariate and multivariate schemes, the odds ratio of hospitalization analyzed by the unconditional and correlated logistic regression showed similar results for 95% confidence intervals. Appendix A Table A3 presents the odds ratio (OR) of hospitalization due to an AEFI only for the correlated logistic regression analysis. Significance in the odds of hospitalization was seen for age classes. Younger children (1–4 years and less than 1 year of age) had higher odds of hospitalization than children 5–9 years of age, and these differences were significant in the univariate model (OR = 1.5 for the first two age classes, while OR = 1.0 for the oldest age class).

When considering the full model accounting for gender, vaccine types, and doses, the difference was no longer significant between children younger than 1 year and between 5 and 9 years (OR = 1.1). Compared with the TETRA vaccine, the YF and ORV vaccines showed higher odds of hospitalization (with or without other factors in the full model, OR = 1.8 and 1.5, respectively). For the DPT and BCG vaccines (OR = 0.8 and 0.7, respectively), a decrease in the chance of hospitalization was observed, when compared to TETRA. When comparing the vaccine doses, adverse events that occurred after the second booster dose had a chance of hospitalization that was significantly lower than for the first booster and third, second, and first doses.

## 4. Discussion

This study profiled the most reported AEFIs in Brazilian children (according to the national registry of adverse events) related to the mandated pediatric vaccines. In Brazil, AEFI surveillance is mandatory and administered by the Ministry of Health. In this study, vaccines were presented as oral and injectable in order to better understand the nature of the Brazilian AEFI. Despite high regional diversity in the rate of AEFIs, the vaccine with the highest reported prevalence of adverse effects were those that were injected (TETRA > DTP > BCG > MMR > YF). The lowest AEFI prevalence was noted for the oral vaccines (ORV > OPV). The AEFI and its prevalence rate depended on the vaccine and age of the child. Because vaccine exposure is skewed towards early age, AEFIs for some vaccines seemed to have a higher incidence in young children.

The differences in age of AEFI varied according to the vaccine. Therefore, the early AEFIs (registered for HB) were considered mild. The percentage of febrile adverse events varied with age for almost all vaccines (Table 2 and Table 3). The percentage of vaccine-induced febrile seizures diminished with age, and these were highest at the age of <1 year and lowest in the 4–9-year-old group. Age-associated outcomes of fever and febrile convulsion were seen for vaccination with HB and TETRA, respectively.

Injectable vaccines (TETRA, DTP, BCG, MMR, YF) showed a distinct AEFI pattern when compared with oral administered vaccines (Table 1). During infancy, breastfeeding can influence vaccine response [15] and it may or may not affect the incidence of adverse behavior, local reactions, and fever [16]. The AEFIs related to the TETRA vaccine in Brazil have been discussed by Monteiro et al. [8]. They found them higher than in other countries and attributed them to the case definition adopted in Brazil which downplays mild events and late onset AEFIs. This probably results in an overestimation of the severe events [8].

Our results concurred with those of the study by Monteiro et al. [8] on DTP between the years of 2002 to 2005. Also, Alguacil-Ramos et al. [17] reported that diphtheria, tetanus, and acellular pertussis (DTaP) had the highest AEFI reported in Spanish children (96.6/100,000 doses). Gkampeta et al. [18] reviewed the association of vaccines with an elevated risk of febrile events. They pointed to seizures after DTP and MMR among the most frequently cited adverse vaccine events in the literature. They suggested a lower risk of seizures with DTP vaccination in the first 2–4 months of life. It seems that the MMR vaccination is not associated with occurrence of seizures in the first year of life. However, delayed vaccination post 15 months is associated with more postvaccination seizures [18].

Thomas et al. [19] reviewed the AEFIs associated with YF vaccines and reported no cases in children and infants in active surveillance studies. In passive surveillance studies, there was a very low rate of adverse events for the general population (0.51 AEFI/million doses). Specific neurological adverse events extracted from this database from 2007 to 2012 have been reported for YF in Brazil [20]. The highest rate of neurological adverse events (0.83 per 100,000 doses) was in the age group from 5 to 9 years.

The relationship between rotavirus vaccine and intussusceptions is known for early vaccines that were withdrawn from the market. Nevertheless, studies from Australia and the United States have shown that new rotavirus vaccines still carry a significant increased risk of intussusception [21]. Indeed, Patel et al. [22] showed that the rotavirus vaccine could have caused additional cases of intussusception in Mexico (approximately 1 per 51,000 infants) and Brazil (approximately 1 per 68,000 infants) per year.

Moylett and Henderson [23] discussed the mechanistic actions of the risks of vaccine administration associated with adverse events—those occurring in the acute setting (like AEFIs) and those related to risk of developing medical conditions in the future [23]. Vaccines are formulated to contain preservatives (Thimerosal) and adjuvants (aluminum), which carry neurotoxic risks and/or unintended immune reactions [24] that can manifest over months or years. In this context, nonspecific effects of vaccines have been gaining traction. Indeed, in most studies, Thimerosal-containing vaccines have shown a high risk of developing tic disorders or contact dermatitis [24]. The current AEFI system does not recognize any of these chronic conditions that may result from or be associated with pediatric vaccines [24].

In certain populations, vaccines can have an opposing nonspecific effect; therefore, interactions of vaccines and AEFI may become a topic of investigation. Goldman and Miller [25] analyzed the Vaccine Adverse Event Reporting System database (of the United States) and suggested a positive correlation between the number of vaccine doses and the rate of hospitalizations and deaths. Vaccine (DTaP/*Haemophilus influenzae* b) associated adverse events (occurring between 2002 and 2005) that evolved to death were 0.2%, as reported by Monteiro et al. [9].

Whitaker et al. [26] reviewed vaccines’ adverse effects taking into consideration “adversomics” as a new paradigm to understand vaccine safety. While such approaches represent the cutting edge of integrative sciences (immunogenomics and system biology), they do not preclude ecological studies of different populations in postvaccine licensure. Indeed, we do not yet have a system suited to understand/capture AEFIs according to the type of vaccine (live attenuated, inactivated whole cell, adjuvanted, monovalent, polyvalent, etc.) or that profiles children’s individual biology.

The limitations of this study mainly appeared due to issues inherent to vaccine adverse events reporting systems. These issues have been discussed elsewhere [27] and apply to the present work. This type of surveillance used in this study is low cost and simple; however, it has the disadvantage of being less sensitive, so it is more vulnerable to subnotification. Therefore, a passive surveillance system carries less representation and presents difficulties in the standardization of case definitions [7]. Summing up, this kind of system is subject to: (a) bias (underreporting of common mild adverse events); (b) quality and completeness of reporting; and (c) reporting efficiency. However, in our case, all the AEFIs reported had confirmation—they all required medical attention and/or hospitalization. Long-lasting effects or disabling illness caused by or associated with the reported AEFI were not followed by the system after the medical visit or hospitalization had taken place. Although the AEFIs were clearly perceived as harmful or needing medical attention, in cases with death as the outcome, the confirmation has to go through a detailed and complex medical procedure that hardly ever gets reported in the Brazilian AEFI. It is also noteworthy that there are no compensation programs in place to assist victims of AEFI. When an AEFI leads to an injury, Brazilian families bear the burden. Cases taken to court are few and concentrated in the most developed parts of the country; furthermore, judicial decisions, when compared, involve contradictory sentences for similar cases [28].

## 5. Conclusions

Due to the large size of the country and the variety of vaccine brands used, it was beyond the purpose of this study to establish the relative safety of any specific vaccine. However, the Brazilian AEFI system is useful to monitor and manage the safety of the pediatric vaccination program. When used properly, this registry is useful to compare the magnitude and certain characteristics of adverse events associated with the mandated pediatric vaccines. In the present case, it is worth mentioning a high association of the TETRA vaccine with hypotonic hyporesponsive episode and febrile convulsion.

## Figures and Tables

**Table 1:** Total of adverse postvaccination events considering single inoculation (SI) and combined inoculation (CI) with other vaccines in the same visit.

Vaccine	Total of Adverse Events		
	SI	CI	SI/CI (%)
TETRA	30315	32362	93.7
DTP	6405	6840	93.6
BCG	3501	3595	97.4
MMR	1284	1567	81.9
HB	934	1513	61.7
YF	886	1009	87.8
ORV	700	1620	43.2
OPV	407	1881	21.6

TETRA: diphtheria, pertussis, tetanus, Haemophilus influenzae type b; DTP: diphtheria, tetanus, pertussis; BCG: Bacillus Calmette–Guerin; MMR: measles, mumps, rubella; HB: hepatitis B; YF: yellow fever; ORV: oral vaccine against rotavirus diarrhea; OPV: oral poliovirus vaccine.

**Table 2:** Frequency of occurrence of the 10 most reported adverse events related to the injectable vaccines by age groups, considering only single inoculation doses.

Event	Age (Years)							
	<1		1–4		5–9		Total	
	N	%	N	%	N	%	N	%
HB								
Abscess hot spot	218	29.18	1	0.13	0	0	219	29.32
Pain, redness and heat	107	14.32	4	0.54	4	0.54	115	15.39
Induration	72	9.64	3	0.4	0	0	75	10.04
Fever ≥ 39.5 °C	69	9.24	1	0.13	0	0	70	9.37
Other severe events	58	7.76	1	0.13	0	0	59	7.9
HR after 2 h	51	6.83	0	0	1	0.13	52	6.96
Fever < 39.5 °C	49	6.56	2	0.27	0	0	51	6.83
Generalized rash	38	5.09	2	0.27	1	0.13	41	5.49
Lump	31	4.15	2	0.27	0	0	33	4.42
Other local reactions	26	3.48	4	0.54	2	0.27	32	4.28
Total	719	96.25	20	2.68	8	1.07	747	100
TETRA								
HHE	8294	30.32	783	2.86	38	0.14	9115	33.32
Fever ≥ 39.5 °C	4589	16.77	539	1.97	23	0.08	5151	18.83
Fever < 39.5 °C	3270	11.95	196	0.72	7	0.03	3473	12.7
Pain, redness, and heat	2559	9.35	179	0.65	14	0.05	2752	10.06
Febrile convulsion	2067	7.56	415	1.52	18	0.07	2500	9.14
Induration	993	3.63	59	0.22	3	0.01	1055	3.86
Other severe events	962	3.52	90	0.33	1	0	1053	3.85
Afebrile seizure	687	2.51	97	0.35	7	0.03	791	2.89
Lump	707	2.58	36	0.13	2	0.01	745	2.72
Generalized rash	648	2.37	69	0.25	5	0.02	722	2.64
Total	24,776	90.57	2463	9	118	0.43	27,357	100
DTP								
Pain, redness, and heat	53	0.97	816	14.87	438	7.98	1307	23.81
Fever	61	1.11	652	11.88	185	3.37	898	16.36
HHE	82	1.49	564	10.28	157	2.86	803	14.63
Fever < 39.5 °C	42	0.77	506	9.22	152	2.77	700	12.75
Febrile convulsion	36	0.66	458	8.34	95	1.73	589	10.73
Induration	16	0.29	234	4.26	73	1.33	323	5.88
Lump	10	0.18	152	2.77	39	0.71	201	3.66
Abscess hot spot	9	0.16	179	3.26	62	1.13	250	4.55
Difficulty walking	6	0.11	180	3.28	52	0.95	238	4.34
Headache and vomiting	6	0.11	124	2.26	50	0.91	180	3.28
Total	321	5.85	3865	70.41	1303	23.74	5489	100
BCG								
Lymphadenopathy	758	23.53	18	0.56	12	0.37	788	24.46
Abscess hot spot	368	11.42	36	1.12	76	2.36	480	14.9
Cold abscess site	382	11.86	62	1.92	23	0.71	467	14.49
Ulcer > 1 cm	276	8.57	29	0.9	42	1.3	347	10.77
Lymphadenitis > 3 cm	332	10.3	4	0.12	5	0.16	341	10.58
Lump	208	6.46	19	0.59	5	0.16	232	7.2
Suppurated lymphadenitis	214	6.64	11	0.34	1	0.03	226	7.01
Lymphadenopathy > 3 cm	132	4.1	8	0.25	2	0.06	142	4.41
Pain, redness, and heat	91	2.82	3	0.09	9	0.28	103	3.2
Other local reactions	85	2.64	4	0.12	7	0.22	96	2.98
Total	2846	88.33	194	6.02	182	5.65	3222	100
MMR								
Generalized rash	44	3.95	399	35.82	19	1.71	462	41.47
Fever < 39.5 °C	17	1.53	149	13.38	11	0.99	177	15.89
Fever ≥ 39.5 °C	9	0.81	142	12.75	5	0.45	156	14
HR after 2 h	5	0.45	84	7.54	3	0.27	92	8.26
HR up to 2 h	1	0.09	30	2.69	5	0.45	36	3.23
Pain, redness, and heat	5	0.45	37	3.32	22	1.97	64	5.75
Other severe events	5	0.45	31	2.78	4	0.36	40	3.59
Febrile convulsion	4	0.36	28	2.51	1	0.09	33	2.96
Mumps	1	0.09	25	2.24	6	0.54	32	2.87
Headache and vomiting	1	0.09	17	1.53	4	0.36	22	1.97
Total	92	8.26	942	84.56	80	7.18	1114	100
YF								
Generalized rash	140	19.31	32	4.41	5	0.69	177	24.41
HR up to 2 h	95	13.1	19	2.62	5	0.69	119	16.41
HR after 2 h	84	11.59	27	3.72	2	0.28	113	15.59
Fever ≥ 39.5 °C	59	8.14	24	3.31	5	0.69	88	12.14
Fever < 39.5 °C	44	6.07	23	3.17	7	0.97	74	10.21
Other severe events	34	4.69	5	0.69	3	0.41	42	5.79
Pain, redness, and heat	27	3.72	5	0.69	3	0.41	35	4.83
Meningitis	5	0.69	9	1.24	16	2.21	30	4.14
Headache	7	0.97	4	0.55	13	1.79	24	3.31
Headache and vomiting	15	2.07	5	0.69	3	0.41	23	3.17
Total	510	70.34	153	21.1	62	8.55	725	100

TETRA: diphtheria, pertussis, tetanus, Haemophilus influenzae type b; DTP: diphtheria, tetanus, pertussis; BCG: Bacillus Calmette–Guerin; HB: hepatitis B; HHE: hypotonic hyporesponsive episode; HR: hypersensibility reaction; MMR: measles, mumps, rubella; YF: yellow fever.

**Table 3:** Frequency of occurrence of the 10 most reported adverse events related to the oral vaccines by age groups, considering only single inoculation doses.

Event	Age (Years)							
	< 1		4-Jan		9-May		Total	
	N	%	N	%	N	%	N	%
OPV (Oral poliovirus)								
Other severe events	31	9.78	30	9.46	0	0	61	19.24
Generalized rash	21	6.62	28	8.83	1	0.32	50	15.77
Fever ≥ 39.5 °C	13	4.1	25	7.89	0	0	38	11.99
Fever < 39.5 °C	13	4.1	25	7.89	0	0	38	11.99
HR up to 2 h	15	4.73	17	5.36	0	0	32	10.09
HR after 2 h	12	3.79	24	7.57	1	0.32	37	11.67
HHE	12	3.79	3	0.95	0	0	15	4.73
Other local reactions	7	2.21	7	2.21	0	0	14	4.42
Febrile convulsion	4	1.26	10	3.15	0	0	14	4.42
Headache and vomiting	3	0.95	15	4.73	0	0	18	5.68
Total	131	41.32	184	58.04	2	0.63	317	100
ORV (Oral rotavirus)								
Other severe events	329	48.24	3	0.44	0	0	332	48.68
Intussusception	173	25.37	3	0.44	1	0.15	177	25.95
Other local reactions	43	6.3	1	0.15	1	0.15	45	6.6
Fever ≥ 39.5 °C	35	5.13	0	0	0	0	35	5.13
Headache and vomiting	33	4.84	1	0.15	0	0	34	4.99
Fever < 39.5 °C	32	4.69	0	0	0	0	32	4.69
HR after 2 h	9	1.32	0	0	0	0	9	1.32
Generalized rash	7	1.03	0	0	0	0	7	1.03
Angioedema	7	1.03	0	0	0	0	7	1.03
HHE	4	0.59	0	0	0	0	4	0.59
Total	672	98.53	8	1.17	2	0.29	682	100

TETRA: diphtheria, pertussis, tetanus, Haemophilus influenzae type b; DTP: diphtheria, tetanus, pertussis; BCG: Bacillus Calmette–Guerin; HB: hepatitis B; HHE: hypotonic hyporesponsive episode; HR: hypersensibility reaction; MMR: measles, mumps, rubella; YF: yellow fever.

## Appendix A

**Table A1:** Adjusted residuals analysis for association estimates between vaccines and the 10 most reported adverse events (considering only single inoculation doses).

AEFI	Vaccine								
	TETRA	DTP	BCG	OPV	MMR	ORV	HB	YF	*p*
Test 1									<0.0001
HHE									
Yes	9115 (38.5%)	803 (16.6%)	1 0.00%	15 (4.4%)	14 (1.4%)	4 −0.60%	30 (3.9%)	10 (1.4%)	
	[60.4]	[−19.5]	[−37.6]	[−9.9]	[−19.1]	[−15.8]	[−15.2]	[−16.1]	
No	14,580 (61.5%)	4037 (83.4%)	3252 (100.0%)	327 (95.6%)	975 (98.6%)	643 (99.4%)	740 (96.1%)	699 (98.6%)	
	[−60.4]	[19.5]	[37.6]	[9.9]	[19.1]	[15.8]	[15.2]	[16.1]	
Total	23,695 (100.0%)	4840 (100.0%)	3253 (100.0%)	342 (100.0%)	989 (100.0%)	647 (100.0%)	770 (100.0%)	709 (100.0%)	
Test 2									<0.0001
Afebrile seizure	2724 (11.5%)	599 (12.4%)	0 0.00%	18 (5.3%)	34 (3.4%)	4 −0.60%	23 (3.0%)	13 (1.8%)	
	[16.4]	[6.8]	[−19.6]	[−2.8]	[−6.7]	[−7.9]	[−6.4]	[−7.1]	
Febrile convulsion	562 (2.4%)	92 (1.9%)	0 0.00%	1 −0.30%	7 −0.70%	2 −0.30%	5 −0.60%	7 −1.00%	
	[8.9]	[−0.1]	[−8.4]	[−2.2]	[−2.8]	[−3.0]	[−2.6]	[−1.8]	
Fever without seizure	8040 (33.9%)	1502 (31.0%)	44 (1.4%)	74 (21.6%)	326 (33.0%)	65 (10.0%)	116 (15.1%)	155 (21.9%)	
	[27.4]	[2.9]	[−36.7]	[−3.1]	[2.6]	[−10.9]	[−8.8]	[−4.4]	
Cases without	12,369 (52.2%)	2647 (54.7%)	3209 (98.6%)	249 (72.8%)	622 (62.9%)	576 (89.0%)	626 (81.3%)	534 (75.3%)	
fever or convulsion	[−37.8]	[−6.7]	[48.1]	[5.2]	[2.5]	[15.6]	[12.7]	[8.9]	
Total	23,695 (100.0%)	4840 (100.0%)	3253 (100.0%)	342 (100.0%)	989 (100.0%)	647 (100.0%)	770 (100.0%)	709 (100.0%)	
Test 3									<0.0001
Generalized rash									
Yes	722 (3.0%)	112 (2.3%)	7 −0.20%	50 (14.6%)	462 (46.7%)	7 −1.10%	41 (5.3%)	177 (25.0%)	
	[−18.6]	[−7.8]	[−12.3]	[9.1]	[65.1]	[−4.2]	[1.1]	[26.6]	
No	22,973 (97.0%)	4728 (97.7%)	3246 (99.8%)	292 (85.4%)	527 (53.3%)	640 (98.9%)	729 (94.7%)	532 (75.0%)	
	[18.6]	[7.8]	[12.3]	[−9.1]	[−65.1]	[4.2]	[−1.1]	[−26.6]	
Total	23,695 (100.0%)	4840 (100.0%)	3253 (100.0%)	342 (100.0%)	989 (100.0%)	647 (100.0%)	770 (100.0%)	709 (100.0%)	
Test 4									<0.0001
Increased lymph nodes									
Yes	1079 (4.6%)	345 (7.1%)	1513 (46.5%)	4 (1.2%)	38 (3.8%)	0 0.00%	82 (10.6%)	10 (1.4%)	
	[−39.7]	[−4.2]	[80.2]	[−5.0]	[−5.5]	[−7.9]	[1.9]	[−7.0]	
No	22,616 (95.4%)	4495 (92.9%)	1740 (53.5%)	338 (98.8%)	951 (96.2%)	647 (100.0%)	688 (89.4%)	699 (98.6%)	
	[39.7]	[4.2]	[−80.2]	[5.0]	[5.5]	[7.9]	[−1.9]	[7.0]	
Total	23,695 (100.0%)	4840 (100.0%)	3253 (100.0%)	342 (100.0%)	989 (100.0%)	647 (100.0%)	770 (100.0%)	709 (100.0%)	

**Note:** The value between brackets [] corresponds to the adjusted residuals.

**Table A2:** Results of multiple comparisons of vaccines for median age (in months) of affected children with the 10 most reported adverse events using the Dunn hypothesis test, considering only single inoculation doses.

Vaccine	Median Age (in Months)	Homogeneous Groups *
DTP	19	A
OPV	16	B
MMR	13	B
YF	10	B
TETRA	5	C
ORV	4	D
BCG	3	D
HB	2	E

**Note:** * Different letters represent significantly different medians (α = 0.05).

**Table A3:** Odds ratio (OR) of hospitalization for adverse postvaccination cases and the 95% confidence interval in the univariate and multivariate models using the studied predictors, considering a logistic regression model for correlated data (GEE) for single inoculation doses.

Predictor	Univariate		Multivariate	
	OR	IC 95%	OR	IC 95%
Gender				
Female	1	-	1	-
Male	1.0	(0.99; 1.08)	1.0	(0.99; 1.08)
Age				
5–9 years	1	-	1	-
1–4 years	1.5	(1.38; 1.71)	1.2	(1.09; 1.39)
<1 year	1.5	(1.35; 1.65)	1.1	(0.98; 1.27)
Vaccine				
TETRA	1	-	1	-
DTP	0.8	(0.76; 0.86)	0.8	(0.69; 0.89)
BCG	0.7	(0.65; 0.76)	0.7	(0.67; 0.79)
OPV	1.1	(0.91; 1.43)	1.3	(0.99; 1.63)
MMR	1.0	(0.84; 1.08)	0.9	(0.79; 1.07)
ORV	1.8	(1.48; 2.11)	1.8	(1.52; 2.16)
HB	1.0	(0.90; 1.21)	1.1	(0.91; 1.22)
YF	1.4	(1.21; 1.68)	1.5	(1.25; 1.74)
Applied Dosage				
2nd booster	1	-	1	-
1st booster	1.5	(1.37; 1.73)	1.5	(1.31; 1.70)
3rd dose	1.7	(1.52; 1.88)	1.4	(1.19; 1.62)
2nd dose	1.6	(1.42; 1.74)	1.3	(1.13; 1.52)
1st dose	1.5	(1.33; 1.61)	1.3	(1.09; 1.46)

TETRA: diphtheria, pertussis, tetanus, Haemophilus influenzae type b; DTP: diphtheria, tetanus, pertussis; BCG: Bacillus Calmette–Guerin; MMR: measles, mumps, rubella; HB: hepatitis B; YF: yellow fever.
